# Global Bibliometric Developments on Solid Waste Recycling in Concrete Construction Engineering

**DOI:** 10.3390/ma15124142

**Published:** 2022-06-10

**Authors:** Xiaoshan Zhang, Yue Xiao, Yongjie Xue, Jian Liu, Zongwu Chen, Ronghui Zhang

**Affiliations:** 1 State Key Laboratory of Silicate Materials for Architectures, Wuhan University of Technology, Wuhan 430070, China; zxs97@whut.edu.cn (X.Z.); xiaoy@whut.edu.cn (Y.X.); xyjskl@whut.edu.cn (Y.X.); liujian1997@whut.edu.cn (J.L.); 2 Key Laboratory of Road Structure and Material of Ministry of Transport, Changsha University of Science & Technology, Changsha 410114, China; chenzw@cug.edu.cn; 3 Faculty of Engineering, China University of Geosciences (Wuhan), Wuhan 430074, China; 4 Library of Wuhan University of Technology, Wuhan University of Technology, Wuhan 430070, China

**Keywords:** solid waste, cement, bibliometric analysis, fly ash

## Abstract

The precise and visual analysis of solid waste recycling in concrete construction engineering is critical for the development of ecological civilization and for the secure supply of resources. This research makes a bibliometric analysis of the solid waste application in concrete construction engineering from 2000 to 2021 based on the Web of Science. The global bibliometric status, current research focus and future directions were used to indicate the global development of solid waste recycling in concrete construction engineering. The most reused solid wastes and most solid waste productive regions were concluded with this bibliometric analysis. China is far ahead of other countries in solid waste recycling in all aspects and heavy metal is one of the most prominent solid waste themes in China. By analyzing the most studied solid waste, fly ash appears to be the most popular and is widely used; half of the top ten-cited papers are correlated with it.

## 1. Introduction

The growing urbanization, limited resources, and improvements in material science have necessitated an immediate solution to the solid waste problem. Throughout the twentieth century, scholars from several countries conducted extensive studies on various aspects of solid waste [1,2,3,4], making solid waste application a hot issue globally. In general, solid waste has a huge value in terms of extraction and usage. China generates 600 million tons of industrial waste residue and waste ore each year, which includes a large number of metals, rare metals, and construction materials that may still be mined and utilized [5]. Making full use of solid waste in road construction not only solves the issue of solid waste accumulation, but also reduces the consumption of road materials, enhances road performance, and extends the life of the road [6,7,8].

There are five categories of solid waste that are widely used in construction engineering, including municipal solid waste (MSW), sludge, food waste, electrical waste and construction and demolition waste [9]. Li et al. [10] employed bottom ash as a supplemental cementing ingredient in the preparation of blended cement and discovered that when municipal solid waste incineration (MSWI) bottom ash proportion was less than 30%, the strength of the blended cement could reach the standard. Xue et al. [11] utilized MSWI fly ash as a partial substitution for fine aggregate or mineral filler in a stone matrix asphalt (SMA) mixture, which saves natural rock completely and promotes a solid waste utilization rate of over 90%. Yao and colleagues [12] evaluated the impact of replacing cement and sand (0–25%) with fly ash (FA) on the mechanical and shrinkage parameters of rapid hardening Portland cement (RHPC) by using the orthogonal design technique and shrinkage testing, and increased FA was reported to improve dry shrinkage and plastic shrinkage behavior. There are numerous similar kinds of literature that may be categorized and statistically evaluated in order to properly detect various solid wastes at different times [13,14]. Scholars in related disciplines will be able to choose the types and recycling treatment of solid waste in the future based on the application in various fields and countries.

The bibliometric analysis approach can swiftly count solid waste-related publication data from various years, journals, institutions and nations, and apply analytical methods from several databases to reuse the solid waste in the field of construction engineering [15]. In 2007, Huang et al. [16] performed a statistical analysis on solid waste re-application in asphalt pavements, where waste glass, steel slag, tyres, and plastics are the most common solid wastes involved. Siddique R [17,18,19,20] introduced the physical and chemical characteristics, and mineral and element composition of MSW ash and cement kiln dust (CKD) in detail and analyzed their effect on the performance of concrete or cement mortar through the bibliometric method. Furthermore, literature econometric analysis was utilized to investigate sulfate removal [21,22,23,24], slag and sludge usage [25,26,27,28], reusing fly ash or sulfate to consolidate heavy metal-polluted soil [29,30,31,32], and other topics. Many investigations on various solid wastes were undertaken using bibliography analysis, but all forms of information in the articles will change over time, necessitating the resolution of solid waste statistics in concrete construction engineering.

This research makes a bibliometric analysis of the solid waste application in concrete construction engineering in the past twenty years leading up to 2021, and thoroughly explains the variation in output, which was then used to present the global bibliometric status of solid waste recycling. The most reused solid wastes and most solid waste productive regions were then discussed and concluded following the presented bibliometric analysis.

## 2. Methodology and Data Resource

### 2.1. Data Resources

Web of Science Core Collection was considered an accurate engine search method and perfect graphic display in Web of Science, which ranks at the forefront of world database usage. Therefore, this study makes full use of this database and uses Science Citation Index Expanded (SCI) and Social Sciences Citation Index (SSCI) for a more detailed search of science categories. Publications were retrieved from 2000 to 1 January 2022 with the key search terms “Topic—solid waste” and “TITLE—cement”, which means the records of publications containing the solid waste in the abstract, title, keywords and/or the article, and the cement in the title. The final engine search results show 802 articles related to the selection criteria. These articles were preliminarily classified by the web of science, then were analyzed and sorted from different statistical aspects such as productive country or institute, keywords and so on from different data analysis methodologies.

### 2.2. Methodology

VOSviewer 1.6.17 (Developed by Leiden University’s Centre for Science and Technology Studies (CWTS), Leiden, The Netherlands) can be used to generate and visualize a co-occurrence network of relevant phrases taken from scientific literature, which creates network maps to establish connections and evaluate keywords, institutions, and other entities. Web of Science, Scopus, Dimensions, Lens and PubMed are among the databases it can examine. Zooming and scrolling, density (a quick overview of the main areas) and overlay visualizations (show developments over time), screenshots can be adapted as a tool to visualize the different data. Advanced layout and clustering techniques, natural language processing techniques and bibliometric networks are the characteristics of the software. In this paper, VOSviewer is used to create co-occurrence networks on related keywords. The layout and clustering results can be obtained by importing and fine-tuning each parameter. 

## 3. Results Analysis

### 3.1. General Trends

**Figure 1:**
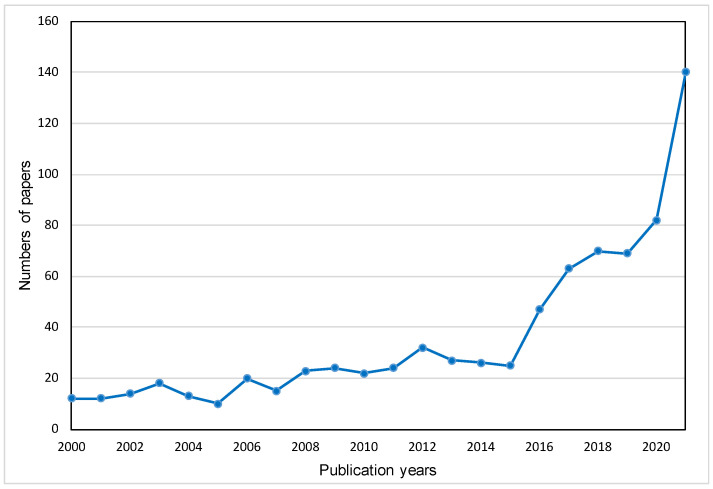
The trends of annual publications from 2000 to 2021.

Figure 1 depicts the annual number of publications between 2000 and 2021. The number of publications climbed somewhat in the years preceding 2015, although the range was small, accounting for less than 5% of the total. The first paper, published in the Journal of *Thermal Analysis and Calorimetry* in 2000 [33], investigated the thermal stability of radioactive solid waste containing NaNO_3_ in cement and bitumen. Another of the three oldest papers all focused on leaching tests of solid waste recycling, containing leaching of borate waste cement matrices [34], full-scale leach tests performed on cemented and concrete waste [35] and the leaching process on cement-stabilized air pollution control (APC) residues from MSWI [36]. Furthermore, the number of papers climbed rapidly in 2016 with an increment of up to 50%, indicating that the research on solid waste in cement received more attention starting from this year and the publishing trends remained comparable during the three years from 2017 to 2019. From the end of 2019 to now, solid waste-related articles have increased significantly, among which the publications in 2020 and 2021 accounted for the largest proportion of 10.33% (82 papers) and 17.63% (140 papers), respectively.

### 3.2. Global Bibliometric Status

The global development of reusing solid waste in concrete construction engineering was evaluated by countries and institutes according to their peer-reviewed publications.

#### 3.2.1. Country Statistics of Solid Waste Recycling

**Figure 2:**
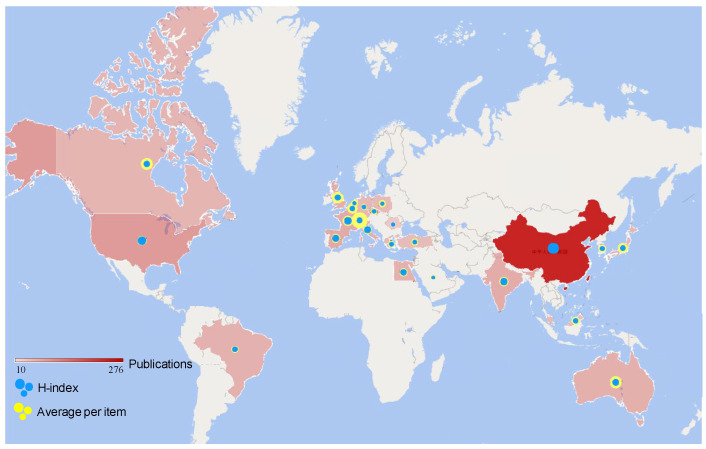
Publication distribution map of the top twenty-five productive countries.

The publication distribution map of the top 25 productive countries/territories is displayed in Figure 2, revealing that China was the leading publishing country with 282 papers, much outnumbering the 66 papers published in the USA, then the leader countries were followed closely by Spain (44 papers), France (39 papers), India (37 papers), Italy (35 papers), the UK (34 papers) and Australia (31 papers) all with more than 30 publications each. The USA attached much more importance to solid waste as a developed country in the early 1970s [37]. China has quickly risen throughout the twentieth century, the usage of solid waste, particularly solid waste in cement, prompting the related articles from China to take first place, as the citations have increased proportionately and reached a new high of 1890 in 2021 (85 papers).

As a method of evaluating academic achievement, the H-index is of great guiding significance in the field of science [38]. The size of blue bubbles in Figure 2 shows the level of the H-index in different countries. The higher the H-index, the greater influence of the paper, thus the top five countries on the H-index were China (41), the USA (23), France (20), India (18) and Spain (17).

The average per item is the main index for citation analysis of sci-tech journals, which is defined as the arithmetic average of the number of citations per article in the journal. As a consequence, the average per item is an important means to evaluate the effect of an article on other research papers, which is shown as yellow bubbles in Figure 2. Although the total number of papers published by China far exceeds that of other countries, the average per item (20.62) is far behind, such as Switzerland (79.82), Australia (46.31), Canada (45.55), and the UK (43.67). The USA, which ranks second in terms of the number of posts, also has an average of 27.76. The reason is that the average per item contains the citation of himself/herself to this paper. When the overall number of publications in a nation is insufficient, the citations that accompany them have an impact on the final average per item. The H-index and average per item of the top fifteen countries with published articles are shown in Table 1.

**Figure 3:**
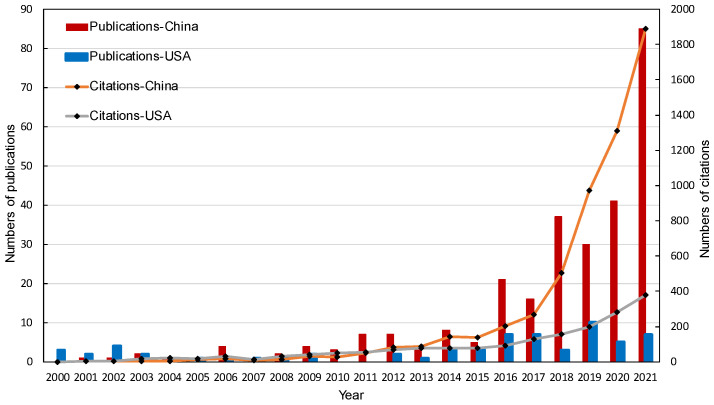
Numbers of publications and citations of China and the USA.

Figure 3 depicts a comparison of publication volume and annual citations between China and the USA over time (the top two countries in publication volume). The number of publications and citations in both countries did not alter significantly in the early 21st century when they were at a low point. It did not considerably increase until after 2015. In the last 5 years, the citations of China have increased proportionately and reached a new high of 1890 in 2021 (85 papers), indicating the expanding worldwide importance of China, while the USA has gradually climbed, reaching a maximum of 380 in 2021 (7 Papers). This phenomenon also demonstrates that as civilization has progressed, solid waste has received more attention.

**Figure 4:**
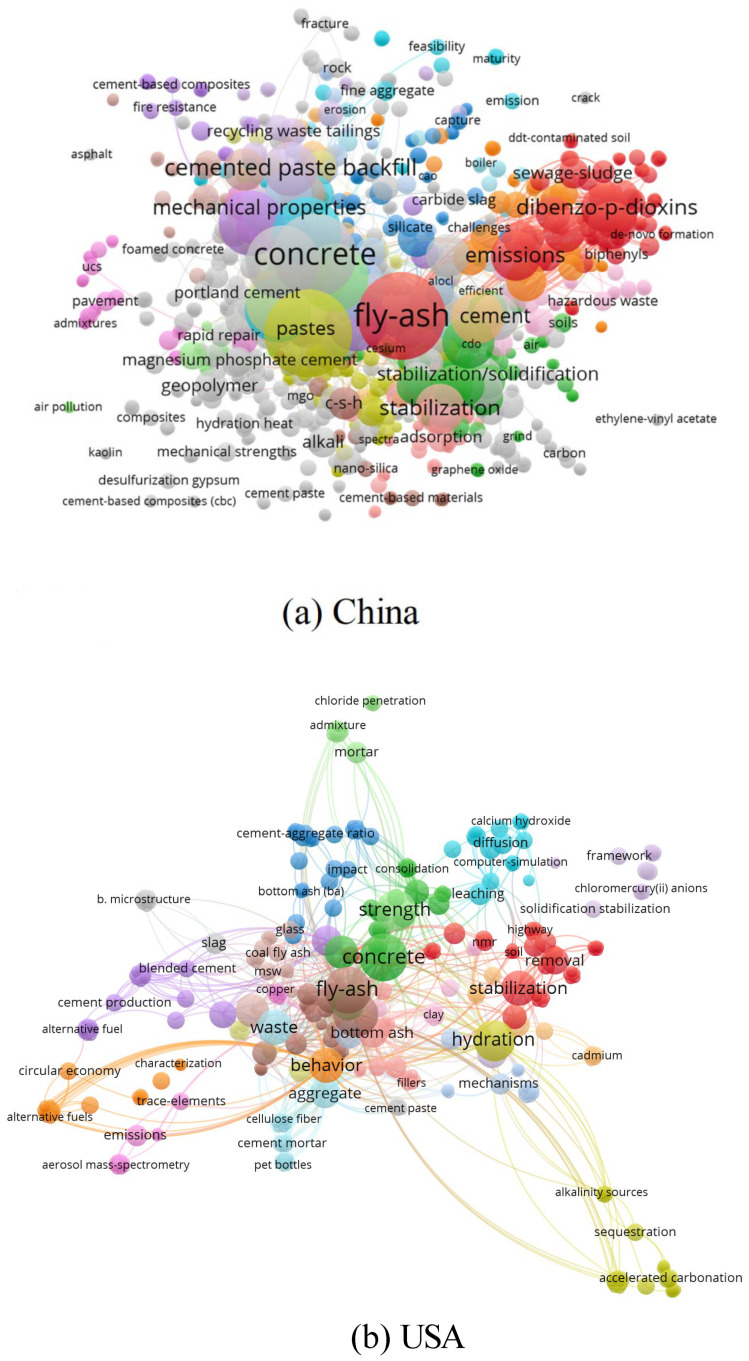
Keywords network visualization graphics of (**a**) China and (**b**) the USA.

VOSviewer was used to analyze the characteristics of 282 papers in China and 66 papers in the USA, respectively, and the network visualization graphics of 1473 and 442 keywords are demonstrated in Figure 4. Fly ash and concrete are still the most focused-on recycling materials for solid waste utilization in these two countries. Cement paste and emission are two crucial phrases in the solid waste treatment process for China, hence they prefer to concentrate on the way of solid waste reuse and environmental effect. For the USA, they pay more attention to the strength, hydration effect and stabilization. Heavy metal is one of the most prominent solid waste themes in China according to the analysis of different articles, with copper, zinc, lead, lithium slag, nickel slag, mercury-contaminated hazardous wastes, and other materials solidified/stabilized in various types of cement. Three of the top five-cited papers are about heavy metal waste. The work with the most citations [39], is a review on the significance of heavy metal waste in cement, which explored the interaction between the cement phase and heavy metals, the solidification process, and the governing variables of cement-based stability. Li [40] stabilized high-concentration copper, zinc, and lead sludge using fly ash (FA) and ordinary Portland cement (OPC), which not only solidified heavy metal waste but also repurposed fly ash. Aside from heavy metals research, the primary objectives of construction solid waste recycling in China’s mining include various fly ash, tailings, red mud, and coal gangue utilized in cement paste backfill, cement mortar, cement clinker, and so on. 

#### 3.2.2. Institution Statistics of Solid Waste Recycling

**Figure 5:**
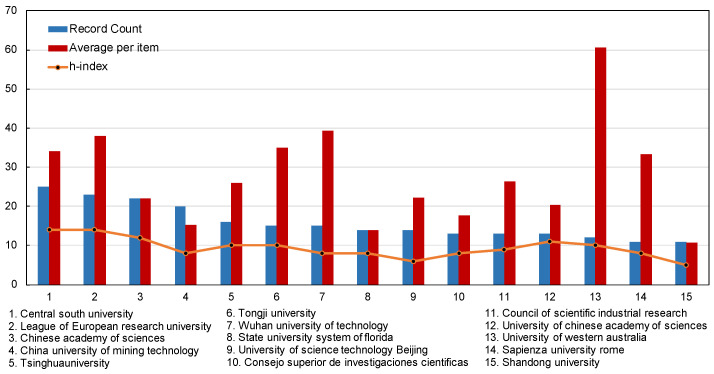
Growth trends of the top fifteen most productive institutions.

Figure 5 presents the top fifteen most productive institutions all over the world according to their publications, most of which are from China. The Central South University tied for first place with an output of 25, while the League of European Research Universities (23 papers) followed closely with the same H-index of 14, respectively. The Chinese Academy of Sciences (22 papers), The China University of Mining Technology (20 papers), Tsinghua University (16 papers), Tongji University (15 papers) and The Wuhan University of Technology (15 papers) are all from China, which were ranked from the third to seventh in the most productive institutions. Among them, Tongji University and the Wuhan University of Technology tied for sixth place. These institutions are all well-known in the field of road construction in the world and play a significant constructive role in solid waste management.

The H-index of each institution is not significantly different, as shown in Table 2. The Central South University, League of European Research Universities and Chinese Academy of Sciences still rank in the top three, while most of the other institutions in the H-index fluctuate between 8 to 10. The University of Western Australia, along with the Wuhan University of Technology, ranked No. 2 and the League of European Research Universities, ranked No. 3, are the world’s top universities specializing in materials and have world-class research facilities in the field of road construction, with an average per item of 60.58.

### 3.3. Research Focus and Future Directions

#### 3.3.1. The Most Studied Solid Waste

There are many kinds of solid waste in the world, such as fly ash, steel slag, blast furnace slag, red mud, coal gangue, coal cinder, sulfuric slag, waste gypsum, desulfurized ash, carbide slag, brine sludge and so on. According to the bibliometric analysis of 802 articles, it is found that fly ash is the most used with 305 articles, followed by blast furnace slag (72), waste gypsum (40), steel slag (27), red mud (20), etc. The statistics of various solid waste research contents can better facilitate other scholars to sort out and evaluate data in order to identify better research objectives.

Table 3 summarizes the number of articles and the top ten countries on the application of fly ash in cement during the past decade. The number of research articles on fly ash should have gradually grown with the passage of time during the recent decade and they reach a new high of 43 articles in 2021. China remained the most productive country accounting for 44.26% of the total with 135 articles published, well ahead of the USA, which had 35 articles (11.47%) and was in second place. In addition, there were 276 articles about solid waste published in China (shown in Table 1 and Figure 2), of which fly ash accounted for 48.9% of the total. The articles regarding fly ash published in the USA also account for 53% (a total of 66 papers), indicating that fly ash is developing rapidly all over the world and is a key execution target of solid waste development.

#### 3.3.2. Keyword Analysis

**Figure 6:**
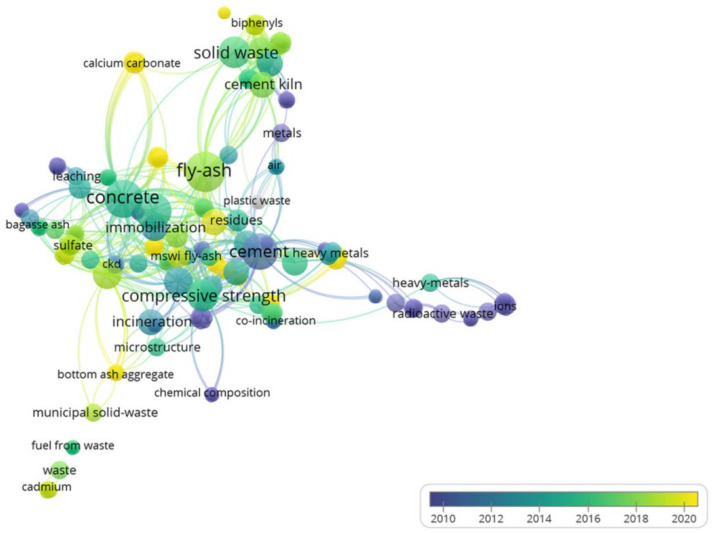
Keywords overlay visualization graphics of publications.

The connected keywords are put together according to the software—VOSviewers and the largest set of connected items consists of 286 items, which is shown in Figure 6. The darker the color of the keyword (close to purple), the older the year is; the bigger the ball, the stronger the link strength of the keyword. The top ten keywords with the highest frequency are fly ash (113), hydration (95), concrete (82), compressive strength (74), solid waste (67), cement (60), behavior (53), dibenzo-p-dioxins (51), Portland cement (50) and clinker (49), which indicates that these keywords are the research hotspots in the application field of solid waste in cement. It is worth mentioning that the fundamental feature of diverse solid wastes repurposed from construction materials is compressive strength (74).

Standardized data processing of VOSviewer divides 286 keywords into 19 clusters, and this allocation method is rather complicated. Six clusters are obtained after deleting abbreviations, repetitive words, and singular and plural forms, which are shown in Table 4. The first cluster is mainly a variety of terms related to solid waste and comprises the most different types of keywords. Fly ash still has the strongest total link strength (115). The second and third keywords are the generic terms of solid waste (67) and municipal solid waste (45). Due to its refractory composition and the wide use of raw products, the production of municipal solid waste increased dramatically [41].

The average link strength of the second keywords cluster is the highest, mostly for some construction materials utilized in solid waste conversion, including concrete (82), cement (60), Portland cement (50), clinker (49) and so on. The stronger the link strength of materials, the hotter the research in this area. The other five clusters contain performance characteristics, impact elements, treatments, synthetic products and others, and the link strength of hydration is as high as 95, indicating that hydration is involved in the majority of solid waste treatment methods.

#### 3.3.3. Top-Cited Papers

The citations of an article show its contribution to the application of solid waste in cement. The more citations an article receives, the more scholars identify it and the more weight it is given, which is a positive way to assess a researcher or even an article. Table 5 shows various information about the top ten-cited papers, three of them [39,42,43] are review papers, while the other seven [6,10,40,44,45,46,47] are research papers. These ten papers were published in nine different journals, namely *Cement and Concrete Research* (twice), *Journal of Hazardous Materials* (thrice), *Waste Management* (twice), *Journal of the American Ceramic Society, Environmental Science & Technology, Journal of Cleaner Production*.

The papers written by Lothenbach Barbara [47], from the European Metal Working Plantmakers Association (WMPA) and Fernandez Bertos M [42], from The University College London ranked in the top two citations of 535 and 528, respectively, far higher than the third of 361. The most cited paper [47], is a research paper published in *Cement and Concrete Research*. The effects of temperature and aluminum on ettringite, monosulfate and monocarbonate in cement were analyzed by means of thermodynamic modeling and calculations, which have a deep influence on the conversion and utilization of solid waste. 

The next most cited paper [42], is a review paper published in the *Journal of Hazardous Materials*. This paper described the influence of accelerated carbonization reaction on the treatment of contaminated soil and put forward the prospect of technology, while this paper has little relationship with solid waste in cement. Similar to the second paper, the third one [43], has little correlation with solid waste but belongs to cement application, which established a new database that can be used to calculate the ultimate hydrate mineralogy from a chemical perspective in the system: CaO–Al_2_O_3_–SiO_2_–CaSO_4_–CaCO_3_–H_2_O. These papers demonstrate the limitations of this analysis method, which can only be avoided through manual screening.

The fourth most-cited paper [39], is about the utilization of heavy metals in cement, while the sixth one [40], namely concerning chemical speciation and leaching behavior of heavy metals in cement, had the same research object, the difference between the two is that the sixth is a research paper which pays more attention to the research method and content, whereas the fourth focuses more on the research summary and belongs to review articles. 

The fifth paper [45], is on the use of solid waste as “green” material to supplement the design of new cement adhesives, the development of one part “Just Add Water” geopolymer formula provides a significant possibility for polymer adhesives to be used in the international road construction field. In comparison to the others, the article rated seventh in citations [44], is a novel research paper that adopted the carbon sequestration action of carbonate solid waste on carbon dioxide to accomplish emission reduction. The latter three most-cited papers [6,10,46] are all about using fly ash in cement, either as a raw material or as a curing agent to help stable other heavy metal solid waste.

#### 3.3.4. Future Directions

The most studied solid waste and the top-cited papers represent the main research content and research objectives in the research field, and their content is extensively cited by international academic peers. As a consequence, we can clearly observe that the thermodynamic characteristics of cement, solidification of heavy metals and recycling of fly ash are the core research contents of solid waste application in cement. More solid waste will undoubtedly be fully utilized in cement in the future, but these studies will play a pivotal role in the development period.

### 3.4. Limitations

Various novel and clear methods were used to analyze all relevant articles in the corresponding research fields, which improved the efficiency of scholars in solid waste recycling. However, the database used in the research was the Web of Science Core Collection and stipulated Science Citation Index Expanded (SCI) and Social Sciences Citation Index (SSCI) as a more detailed scientific classification, which made the literature on the application of solid waste in cement in other databases not included and analyzed. In addition, some of the papers searched for in this research were irrelevant to the research topic, manual selection is time-consuming and laborious, especially since this problem is related to the theme and keywords that are chosen by the author(s) themselves, which is inevitable.

## 4. Conclusions

In this work, bibliometric analysis was used to examine the most recent research hotspots on solid waste utilization in concrete construction engineering over the last 20 years. A neural network diagram was created by using VOSviewer software to exhibit keyword relationships, and a 3D map was used to illustrate worldwide cooperation. According to the discussed results, the following conclusions can be drawn:

1. China and the USA are the top two countries that have made the most effect on recycling solid waste in concrete construction engineering. Bibliometric analysis shows that China is the most productive country with 282 peer-reviewed articles, more than four times the amount of articles produced by the USA. There was little difference in the number of publications and the H-index among the top productive institutions.
2. By comparing t co-occurrence of keywords, it demonstrates that China prefers to concentrate on solid waste reuse and environmental effects while the USA pays more attention to the strength, hydration effect and stabilization.
3. Fly ash is the most widely studied and recycled solid waste in the world. The country with the most publications was China, from which papers discussing fly ash accounted for 48.9% of solid waste publications.
4. Keywords can be divided into seven clusters according to their link strength, which are, respectively, related to solid waste categories, construction materials, performance characteristics, impact elements, treatments, synthetic products and others. The top five keywords with the highest frequency are fly ash (113), hydration (95), concrete (82), compressive strength (74), solid waste (67).
5. The list of most cited papers shows that half of the top ten-cited papers are in connection with fly ash. Industry solid wastes, such as heavy metals were another most focused on solid waste in concrete construction engineering.

## Figures and Tables

**Table 1:** Statistics of related publication in top fifteen countries.

Countries	Number of Papers	H-Index	Average per Item
China	282	41	20.62
USA	66	23	27.76
Spain	44	17	26.93
France	39	20	33.92
India	37	18	28.43
Italy	35	16	23.6
UK	34	13	43.67
Taiwan	31	15	26.61
Australia	29	16	46.31
Brazil	29	11	17.31
Egypt	29	14	22.52
Canada	22	14	45.55
South Korea	21	9	18.48
Japan	19	11	32.68
Germany	18	7	13.06
Totals	729	245	427.45

**Table 2:** Statistics of the top fifteen most productive institutions.

Affiliations	Record Count	H-Index	Average per Item
Central South University	25	14	34.04
League of European Research Universities	23	14	37.96
Chinese Academy of Sciences	22	12	22.09
China University of Mining Technology	20	8	15.25
Tsinghua University	16	10	26
Tongji University	15	10	34.93
Wuhan University of Technology	15	8	39.4
State University System of Florida	14	8	14
University of Science & Technology, Beijing	14	6	22.14
Consejo Superior de Investigaciones Cientificas	13	8	17.77
Council of Scientific Industrial Research	13	9	26.31
University of Chinese Academy of Sciences	13	11	20.23
University of Western Australia	12	10	60.58
Sapienza University, Rome	11	8	33.36
Shandong University	11	5	10.73

**Table 3:** The top ten countries on the application of fly ash in cement during the past decade.

Published Year	Numbers	Published Year	Numbers	Countries	Numbers	Countries	Numbers
2021	43	2016	24	China	135	Australia	12
2020	32	2015	9	USA	35	Italy	12
2019	28	2014	7	Taiwan	26	England	11
2018	39	2013	11	India	15	France	11
2017	29	2012	12	Spain	13	Japan	9
**Totals**		**234**		**Totals**		**279**	

**Table 4:** The keyword statistics of seven clusters and their link strength.

Cluster	Keywords	Link Strength	Cluster	Keywords	Link Strength	Cluster	Keywords	Link Strength
Solid waste categories (434)	fly ash	113	Construction Materials (342)			Performance Characteristics (262)		
	solid waste	67						
	municipal solid waste	45		concrete	82		compressive strength	74
	bottom ash	37		cement	60		behavior	53
	municipal solid waste incineration bottom ash	36		Portland cement	50		performance	48
	residues	33		clinker	49		microstructure	25
	persistent organic pollutants	29		cement kiln	48		solidification	21
	slag	29		aggregate	35		stabilization	21
	tailings	24		cement industry	18		sorption	20
	MSWI bottom	21						
Impact elements (162)			Treatments (184)			Synthetic products (60)	alternative fuels	18
	dibenzo-p-dioxins	51		hydration	95		cemented paste backfill	24
	polychlorinated naphthalenes	43		immobilization	32		solid recovered fuels	18
	phosphogypsum	21		incineration	27	Others (112)	heavy metals	30
	sulfate	24		recycling	30		model	29
	temperature	23					plant	27
							management	26

**Table 5:** The statistics of the top ten most cited papers.

Title	The First Author	Journal	Cited	Publication Year	Country
Thermodynamic modeling of the effect of temperature on the hydration and porosity of Portland cement [47]	Lothenbach, B	CCR	535	2007	Switzerland
A review of accelerated carbonation technology in the treatment of cement-based materials and sequestration of CO_2_ [42]	Fernandez Bertos, M	JHM	528	2004	England
Thermodynamic properties of Portland cement hydrates in the system CaO–Al_2_O_3_–SiO_2_–CaSO_4_–CaCO_3_–H_2_O [43]	Matschei, T	CCR	361	2007	Switzerland
Immobilization of heavy metal in cement-based solidification/stabilization: A review [39]	Chen, QY	WM	341	2009	China
Designing Precursors for Geopolymer Cements [45]	Duxson, P	Journal of the American Ceramic Society	330	2008	UK
Heavy metal speciation and leaching behaviors in cement-based solidified/stabilized waste materials [40]	Li, XD	JHM	274	2001	China
Carbon Dioxide Sequestration in Cement Kiln Dust through Mineral Carbonation [44]	Huntzinger, DN	Environmental Science & Technology	192	2009	USA
Recycling MSWI bottom and fly ash as raw materials for Portland cement [6]	Pan, JR	WM	175	2008	Taiwan
Solidification/stabilization of arsenic-containing solid wastes using portland cement, fly ash and polymeric materials [46]	Singh, TS	JHM	164	2006	India
Utilization of municipal solid waste incineration bottom ash in blended cement [10]	Li, XG	JLCP	157	2012	China

## Data Availability

Data supporting reported results can be found on the Web of Science.
